# Workplace physical environmental factors associated with pulmonary tuberculosis among workers: a scoping review

**DOI:** 10.1016/j.ijregi.2026.101010

**Published:** 2026-09-10

**Authors:** Mochammad Sholehhudin, Lutfi Fajar Nuraidah, Aziz Fajar, Tofan Agung Eka Prasetya, Virasakdi Chongsuvivatwong, Erwin Astha Triyono, Sulvy Dwi Anggraini, Faridha Cahyani, Wihda Silvia Rahmah, Allina Fiftiyani Nurjanah

**Affiliations:** 1 Department of Health, Faculty of Vocational Studies, Universitas Airlangga, Surabaya, Indonesia; 2 Department of Epidemiology, Faculty of Public Health, Universitas Airlangga, Surabaya, Indonesia; 3 Faculty of Advanced Technology and Multidiscipline, Universitas Airlangga, Surabaya, Indonesia; 4 Department of Epidemiology, Faculty of Medicine, Prince of Songkla University, Hatyai, Thailand; 5 East Java Provincial Health Office, Surabaya, Indonesia

**Keywords:** Tuberculosis, Physical environment, Workplace, Tuberculosis in the worker population, Ventilation in the workplace

## Abstract

**Objectives:**

Tuberculosis remains a major global public health problem. Workers may experience an increased risk of pulmonary tuberculosis due to prolonged exposure to physical environmental factors, such as poor ventilation and exposure to dust or silica. It may facilitate airborne transmission of *Mycobacterium tuberculosis*. This study aimed to synthesize current evidence on the association of workplace physical environmental factors with the incidence of pulmonary tuberculosis among worker populations.

**Methods:**

A scoping review was conducted following the Preferred Reporting Items for Systematic Reviews and Meta-Analyses Extension for Scoping Reviews Statement guidelines. Searches were performed in Scopus, WoS, PubMed, ScienceDirect, and Google Scholar. Studies published between 2016 and 2026 examining workplace environmental factors associated with pulmonary tuberculosis were considered. The process involved identification, screening, eligibility assessment, and inclusion stages.

**Results:**

A total of 2267 records were identified. After screening and eligibility assessment, 10 studies met the inclusion criteria. The findings consistently indicated that inadequate ventilation, poor air circulation, workplace crowding, exposure to dust and silica, and unfavorable indoor environmental conditions such as temperature and humidity were associated with increased tuberculosis risk among workers.

**Conclusion:**

Workplace physical environmental conditions are associated with tuberculosis transmission in occupational settings. Strengthening ventilation, controlling occupational dust exposure, and improving workplace infection prevention strategies are essential to reduce tuberculosis risk among workers.

## Highlights

• The workplace physical environment contributed to tuberculosis transmission.
• Poor ventilation and air circulation have been associated with tuberculosis.
• Dust and silica exposure significantly contribute to pulmonary tuberculosis susceptibility.
• Overcrowding facilitates airborne transmission and elevates tuberculosis risk.
• Ventilation system control is supposed to prevent workplace tuberculosis transmission.

## Introduction

Tuberculosis (TB) remains one of the most significant infectious diseases globally and continues to pose a major public health challenge despite decades of control efforts. TB is caused by *Mycobacterium tuberculosis* and is primarily transmitted through airborne droplets when individuals with active pulmonary TB cough, sneeze, or speak. According to the latest global estimates reported in the *Global Tuberculosis Report*, approximately 10.8 million people developed TB worldwide in 2023, and the disease caused about 1.25 million deaths globally, including deaths among people living with human immunodeficiency virus (HIV) [1]. These data highlight that TB continues to be a major global health burden, particularly in low- and middle-income countries where social and environmental determinants strongly influence transmission and disease progression.

The global burden of TB is unevenly distributed geographically. The World Health Organization reported that Southeast Asia alone contributes a substantial share of the global TB burden. Furthermore, the persistence of TB indicates that biomedical interventions alone are insufficient, and broader determinants—including environmental and occupational conditions—must also be addressed to effectively control transmission and reduce incidence rates [2].

Another important characteristic of the global TB epidemic is its high prevalence among the adult and productive-age population. Epidemiological data show that the largest numbers of TB cases occur among adults aged 25-54 years. In 2024, adult men aged 15 years and older accounted for approximately 54% of the estimated TB cases worldwide, while adult women contributed around 35%, indicating that the majority of TB cases occur in adults who are actively engaged in the workforce. It may have consequences that extend beyond health outcomes, including loss of income, reduced workforce productivity, and increased economic burden on households and societies [3].

Multiple factors contribute to the high burden of TB among adults and workers. TB transmission is influenced by biological, behavioral, socioeconomic, and environmental determinants. Conditions such as HIV infection, malnutrition, smoking, alcohol use, poverty, overcrowding, and limited healthcare access may increase susceptibility to TB infection and disease progression. In addition, occupational exposure and poor working conditions can elevate TB risk, particularly in environments where airborne transmission is more likely to occur. These findings emphasize the multifactorial nature of TB and the importance of occupational and environmental approaches in TB prevention strategies [4].

Occupational and workplace-related exposures are increasingly recognized as important determinants of TB transmission among workers. Several occupational sectors, including healthcare, mining, construction, and industry, expose workers to environmental hazards that may increase susceptibility to TB infection. Healthcare workers, for example, have a higher risk of TB due to repeated exposure to patients with active TB, particularly in settings with inadequate infection prevention and control measures. In addition, workplace physical conditions such as poor ventilation, overcrowding, inadequate indoor air quality, temperature, humidity, and exposure to airborne dust can influence the transmission of *M. tuberculosis* [5].

In addition to ventilation and indoor air quality, exposure to occupational dust, particularly silica dust, has been strongly associated with increased TB risk among workers. Silica exposure commonly occurs in industries such as mining, construction, and manufacturing. Long-term inhalation of respirable crystalline silica particles can lead to silicosis, a chronic lung disease characterized by inflammation and fibrosis. Importantly, silicosis significantly increases susceptibility to pulmonary TB, and individuals with silica exposure may develop a condition known as silicotuberculosis [6]. Evidence from systematic reviews indicates that workers exposed to silica dust have substantially elevated risks of developing TB compared with unexposed populations [7].

Understanding the relationship between workplace environmental conditions and TB incidence is essential for developing effective prevention strategies among workers. Scoping reviews provide comprehensive evidence synthesis and help identify existing research gaps related to occupational TB risk. By integrating findings from various occupational settings and geographical regions, this review offers insights into how workplace physical environmental factors contribute to pulmonary TB transmission. Therefore, this study aims to examine the associations of workplace environmental conditions with pulmonary TB incidence among workers and to identify key environmental determinants that may support evidence-based occupational health policies and workplace TB prevention strategies.

## Methods

### Study design and reporting framework

This study employed a scoping review to find the association of workplace physical environmental factors with pulmonary TB among workers. This scoping review was conducted in accordance with the methodological guidance for scoping reviews and reported following the Preferred Reporting Items for Systematic Reviews and Meta-Analyses Extension for Scoping Reviews (PRISMA-ScR) Statement. The research question was formulated using the Population, Exposure, Comparator, and Outcome (PECO) framework to define the eligibility criteria and guide the literature search and study selection process.

### Research question and PECO framework

The research question addressed in this review was:

“What is known about the association between workplace physical environmental factors and pulmonary TB among workers?”

The PECO framework used in this study is presented in Table 1.

**Table 1:** PECO framework of the study.

Component	Description
Population (P)	Workers or working-age adults exposed to workplace environments
Exposure (E)	Physical environmental factors in the workplace (ventilation, indoor air quality, crowding, temperature, humidity, dust, silica)
Comparator (C)	Workers with lower exposure or a better workplace environment
Outcome (O)	Incidence or risk of pulmonary tuberculosis

### Eligibility criteria

Studies identified through the search process were assessed according to predefined inclusion and exclusion criteria.

**a. Inclusion Criteria**

Studies were included in this review if they met the following criteria:

1. Published in international peer-reviewed journals
2. Published between January 1, 2016 and March 31, 2026
3. Written in English
4. Examined workplace physical environmental factors and TB incidence or risk
5. Included working populations or occupational groups

**b. Exclusion Criteria**

Studies were excluded if they:

1. Focused solely on clinical treatment or diagnosis
2. Did not include occupational or workplace environmental variables
3. Were editorials, commentaries, conference abstracts, or case reports
4. Were not accessible in full-text format
5. Were review studies

### Information source

A comprehensive literature search was conducted to identify relevant peer-reviewed studies examining the relationship between workplace environmental factors and TB incidence among workers. The databases included:

1. Scopus
2. Web of Science
3. U.S. National Library of Medicine PubMed
4. ScienceDirect
5. Google Scholar

### Search strategy

The literature search was conducted for studies published between January 1, 2016 and March 31, 2026 to capture earlier studies and the most recent evidence relevant to workplace environmental determinants of TB. Search terms were developed using a combination of keywords and Boolean operators based on the PECO framework. The following example search string was used:

("tuberculosis" OR "pulmonary tuberculosis" OR "TB") AND ("workplace" OR "occupational" OR "worker" OR "occupational exposure" OR "industrial worker" OR "construction worker" OR "factory worker" OR "mining worker") AND ("physical environment" OR "ventilation" OR "lighting" OR "temperature" OR "dust exposure" OR "humidity" OR "pm2.5") AND ("risk" OR "incidence" OR "association")

### Study selection

The study selection process was conducted in accordance with the predefined eligibility criteria, and the screening and selection process was reported using PRISMA-ScR. The process consisted of four main steps:

1. Identification & duplicate removal
  All records identified from the database searches were imported into reference management software to organize citations and remove duplicates.
2. Title and abstract screening
  The titles and abstracts of all remaining articles were screened to assess their relevance to the research question.
3. Full-text assessment
  Full texts of potentially eligible articles were retrieved and assessed against the predefined eligibility criteria.
4. Final inclusion
  Studies that satisfied all eligibility criteria were included in the final scoping review.

The study selection process was conducted independently by four reviewers. Disagreements between reviewers were resolved through discussion and consensus. When consensus could not be reached, a fifth reviewer was consulted to make the final decision. Reasons for exclusion at the full-text stage were documented.

### Data extraction/ charting

Data from the included studies were systematically extracted using a standardized data extraction form. The following information was collected from each study:

1. Author(s), title, and publication year
2. Study design and sample size
3. Country or study location
4. Industrial/work area types
5. Population characteristics
6. Key results and findings of associations
7. Key physical environmental exposures in the workplace

### Data synthesis

A narrative synthesis approach was used to summarize the findings of the included studies. The findings were synthesized by grouping studies according to key environmental exposure categories, including:

1. Descriptive numerical summary: number of studies, publication year, country, study design, industry, exposure category
2. Thematic synthesis by environmental exposure: workplace ventilation and indoor air quality, occupational dust exposure (including silica), workplace crowding and density, and other physical environmental conditions
3. Evidence mapping: environmental factor, then occupational setting, then TB outcome, and final evidence (final result of the study analysis)

**Diagram of PRISMA-ScR flow**

## Results

### Study selection (result of PRISMA)

The study selection process followed the recommendations of the PRISMA-ScR Statement. The detailed screening procedure is presented in the PRISMA flow diagram (Figure 1). During the identification stage, a total of 2267 records were retrieved from five electronic databases, including Scopus (*n* = 1504), Web of Science (*n* = 310), PubMed (*n* = 271), ScienceDirect (*n* = 173), and Google Scholar (*n* = 9). After removing duplicate records, 2045 unique articles remained for further screening.

**Figure 1:**
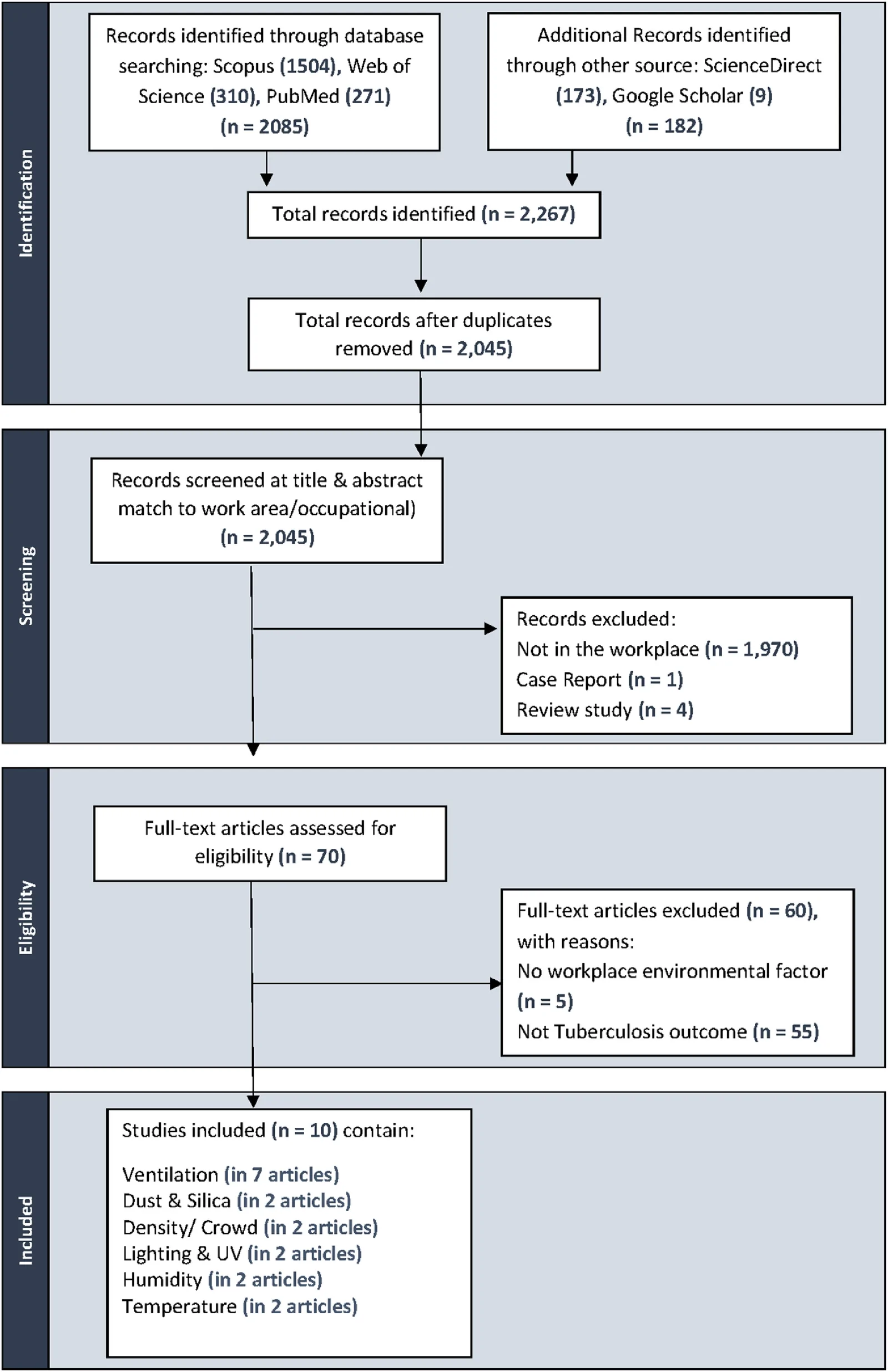
Diagram of the Preferred Reporting Items for Systematic Reviews and Meta-Analyses Extension for Scoping Reviews (PRISMA-ScR) flow.

In the screening stage, the titles and abstracts of the remaining 2045 records were evaluated to determine their relevance to the occupational or workplace context. Following this process, 1975 records were excluded because they were not related to workplace or occupational settings. As a result, 70 articles met the preliminary screening criteria and were carefully assessed against the predefined inclusion and exclusion criteria. A total of 60 articles were excluded at this stage. Five studies did not examine workplace environmental factors, and 55 studies did not report TB as an outcome variable. Finally, 10 studies met all eligibility criteria and were included in the final scoping review.

### Characteristics of included studies

A total of 10 studies fulfilled the inclusion criteria and were included in the final synthesis presented in Table 2. These studies were conducted across several geographical regions with a high burden of TB, highlighting the global relevance of workplace-related risk factors in TB transmission. The included studies originated from countries in Asia, Africa, and America, including Taiwan, India, South Korea, Indonesia, South Africa, Tanzania, Zambia, and USA which are recognized as high TB burden settings.

**Table 2:** Characteristics of exposure assessment.

**No.**	Author	Exposure category and pathway	Specific environmental exposure	Exposure measurement/ assessment	Outcome definition	Main epidemiological findings/effect estimate	Confounders or covariates	Study quality
1	Godfrey et al. [8]	Transmission-related	Ventilation and environmental TB infection control measures	Survey of TB infection control practices at clinical trial sites	TB infection risk among healthcare workers and clinical staff	Inadequate TB control was reported at 22% of sites; improved ventilation and infection control practices were associated with reduced TB infection risk. Specific effect estimate: *P* = 0.02	Administrative control and facility-level measures	High
2	Ngosa and Naidoo [9]	Residential/environmental and individual risk factors	Respirable silica dust exposure	A cross-sectional study of in-service miners. Review of medical data over a 5-year period and cumulative exposure metrics	Silica associated with pulmonary tuberculosis prevalence	High cumulative respirable silica dust category showed a statistically significant association with Pulmonary Tuberculosis. Specific effect estimate: OR = 6.4 (95% CI 1.8-23)	Contact with patients with TB; smoking history	High
3	Jones [10]	Transmission-related	Workplace ventilation systems and TB infection control by respiratory protection	Epidemiological risk analysis. Infection risk was estimated using two dose-response (DR) functions.	Occupationally acquired pulmonary tuberculosis risk among healthcare workers	Healthcare workers were reported to have a higher TB risk than the general population; poor ventilation and exposure to infectious patients with TB were identified as contributing factors. Specific effect estimate: 95% compliance with respiratory protection would eliminate one-third of pulmonary TB infection	Duration of occupational exposure; room volume	Moderate
4	Du CR, [11]	Transmission-related and residential factors	Ventilation, housing conditions, and residential density	measured the effect of improving the ventilation rate on a TB outbreak involving 27 TB cases and 1,665 contacts in poorly ventilated university buildings.	Tuberculosis infection risk	Poor ventilation, poor housing conditions, and high residential density were reported as significant risk factors. Specific effect estimate: *P* <0.001	Low nutritional status	High
5	Kang et al. [12]	Transmission-related	Room ventilation and patient density	Air samples collected from patient rooms; room and environmental characteristics assessed	Airborne TB transmission/environmental contamination	Poor ventilation and overcrowded patient rooms were associated with an increased risk of airborne TB transmission. Air sampling result: *Mycobacterium mageritense and Mycobacterium nontuberculosis* was detected in four of nine air samples from various rooms	Time period of collected samples	Moderate
6	Nandi et al. [13]	Susceptibility-related	Silica dust exposure and duration of exposure	Evaluated chest X–rays at full inspiration. Collected silica in 23 dust samples and quantified by fourier transform infrared spectrophotometer	Pulmonary tuberculosis among mine workers	Silica dust exposure and longer exposure duration were reported to significantly increase TB risk. Specific effect estimate: odds ratio (95% CI) for exposure >10 years to less than 10 years was 4.53 (1.92-10.65)	Age; BMI; smoking; alcohol	High
7	Matuka et al. [14]	Transmission-related	Ventilation, crowd density, humidity, temperature, and UVGI technology	Environmental assessment and collection of 591 air samples from hospital areas	Occupational airborne exposure to *Mycobacterium tuberculosis*	Poor ventilation, high crowd density, and inadequate infection control were reported to increase the risk of TB exposure. Specific effect estimate: *p-value of mechanical ventilation* < 0.013, with OR of 4.77 and 95% CI of 1.398-16.280	Healthcare facilities; seasons; CO_2_	High
8	Mbuya et al. [15]	Susceptibility-related	Silica dust exposure	Observational method for collecting miners' data on site	Tuberculosis among miners and mining communities	Silica exposure was identified as a major TB risk factor; impaired lung function and silicosis were also reported among miners. Specific effect estimate: *P <*0.001	HIV infection; education level	High
9	Adha and Kinanthi [16]	Environmental factors; pathway requires further classification	Humidity, temperature, and ventilation area	Collected primary data on environmental factors	Tuberculosis incidence among farmers	Humidity, temperature, and ventilation area were reported to significantly influence TB incidence. Specific effect estimate: humidity (*P* = 0.007; OR = 3.753), temperature (*P* = 0.032; OR = 3.889), and ventilation area (*P* = 0.025; OR = 3.437)	Age; sex; education; income	Moderate
10	Adha et al. [17]	Environmental factors; pathway requires further classification	Lighting conditions, floor type, and wall type	Collected primary data on environmental factors	Pulmonary tuberculosis among farmers	Lighting was significantly associated with pulmonary tuberculosis., But floor and wall type were not. Specific effect estimate: poor lighting and TB Incidence OR = 3.659 (95% CI: 1.422-9.417)	Age; sex; education; income	Moderate

BMI, body mass index; CI, confidence interval; CO_2_, carbon dioxide; DR, dose-response; HIV, human immunodeficiency virus; OR, odds ratio; TB, tuberculosis; UVGI, ultraviolet germicidal irradiation.

Critical Appraisal and Study Quality Assessment: The methodological quality of the included studies was assessed using the appropriate Joanna Briggs Institute (JBI) Critical Appraisal Tool according to the design of each study (e.g., cross-sectional, cohort, case-control, and analytical observational studies). The appraisal considered methodological domains relevant to each study design, including participant selection, measurement of exposure and outcomes, identification and management of confounding factors, completeness of data, and appropriateness of statistical analyses. For descriptive purposes, study quality was categorized based on the proportion of applicable appraisal criteria fulfilled. Studies meeting 75% of the applicable criteria were classified as high methodological quality, whereas studies meeting 50\05574% of the criteria were classified as moderate methodological quality. The quality appraisal was independently conducted by two reviewers. Any disagreements were resolved through discussion and consensus, and when necessary, a third reviewer was consulted. The resulting quality ratings were incorporated into the evidence tables and considered during the interpretation of the findings.

Most of the included studies employed cross-sectional designs to evaluate the relationship between workplace environmental conditions and TB occurrence among workers. Several studies also used case-control approaches to compare occupational environmental exposures between workers diagnosed with TB and healthy individuals within similar work settings. A smaller number of studies applied cohort designs to assess long-term exposure to workplace environmental factors and their contribution to TB incidence over time. The variation in study design provided complementary evidence regarding occupational exposure and TB risk.

The reviewed studies involved workers from various occupational sectors characterized by substantial environmental exposure. Some studies focuse on mining where exposure to dust and silica particles is common. Other studies examined healthcare workers, who are at increased risk of TB infection because of repeated contact with infectious patients in healthcare facilities. Additional occupational group included agriculture as a workers in informal sectors where humidity, ventilation, temperature, lighting and environmental control systems are often inadequate.

Several workplace physical environmental factors were consistently associated with pulmonary TB incidence. Poor ventilation and inadequate airflow were among the most frequently reported determinants, as they may increase the accumulation and transmission of airborne *M. tuberculosis*. Other reported factors included insufficient lighting, overcrowded workplaces, dust and silica exposure, as well as unfavorable temperature and humidity conditions. These are the titles of selected studies:

1. A Survey of Tuberculosis Infection Control Practices at NIH/NIAID/DAIDS-Supported Clinical Trials Sites in Low- and Middle-Income Countries [8]
2. The Risk of Pulmonary Tuberculosis in Underground Copper Miners in Zambia Exposed to Respirable Silica: A Cross-Sectional Study [9]
3. Burden of Occupationally Acquired Pulmonary Tuberculosis Among Healthcare Workers in the USA: A Risk Analysis [10]
4. Effect of Ventilation Improvement During a Tuberculosis Outbreak in Under-Ventilated University Buildings [11]
5. Detection of Airborne Bacteria from Patient Spaces in Tuberculosis Hospital [12]
6. Pulmonary Tuberculosis Among Stone Miners of India vis-à-vis Silica Exposure [13]
7. Occupational Risk of Airborne *Mycobacterium tuberculosis* Exposure: A Situational Analysis in a Three-Tier Public Healthcare System in South Africa [14]
8. Prevalence and Factors Associated with Tuberculosis Among the Mining Communities in Mererani, Tanzania [15]
9. The Influence of Humidity, Temperature, and Ventilation Area on the Incidence of Tuberculosis Among Farmers in Indonesia: A Case-Control Study [16]
10. The Influence of Lighting, Floor Type, and Wall Type on Pulmonary Tuberculosis in Farmers [17]

### Synthesis of findings

#### Geographical synthesis

The geographical distribution of the included studies was heterogeneous, with evidence originating from Africa, Asia, North America, and Latin America (Table 3). The studies covered a range of occupational sectors, including healthcare, mining, agriculture, and clinical research facilities. Healthcare and mining settings represented the most frequently identified occupational environments.

**Table 3:** Geographical synthesis.

No.	Year	Title	Author	Study Design	Country	Region	Occupational sector	Environmental setting	Exposure location	Spatial scale
1	2016	A Survey of Tuberculosis Infection Control Practices at NIH/NIAID/DAIDS-Supported Clinical Trials Sites in Low- and Middle-Income Countries	Godfrey et al. [8]	Observational Study	Multiple countries	Africa, Asia, Latin America	Healthcare	Clinical facilities	Workplace	Facility
2	2016	The Risk of Pulmonary Tuberculosis in Underground Copper Miners in Zambia Exposed to Respirable Silica: A Cross-Sectional Study	Ngosa et al. [9]	Cross-Sectional Study	Zambia	Africa	Mining	Underground mine	Workplace	Individual/worksite
3	2017	Burden of Occupationally Acquired Pulmonary Tuberculosis Among Healthcare Workers in the USA: A Risk Analysis	Jones [10]	Observational Study	USA	North America	Healthcare	Hospitals/clinics	Workplace	Facility
4	2020	Effect of Ventilation Improvement During a Tuberculosis Outbreak in Under-Ventilated University Buildings	Du CR	Retrospective cohort study	Taiwan	East Asia	University	Institutional buildings	Workplace	Building/facility
5	2020	Detection of Airborne Bacteria from Patient Spaces in Tuberculosis Hospital	Kang et al. [12]	Experimental Study	South Korea	East Asia	Healthcare	TB hospital	Workplace	Facility
6	2020	Pulmonary Tuberculosis Among Stone Miners of India vis-à-vis Silica Exposure	Nandi et al. [13]	Cross-Sectional Study	India	South Asia	Mining	Mining environment	Workplace	Worksite
7	2021	Occupational Risk of Airborne *Mycobacterium tuberculosis* Exposure: A Situational Analysis in a Three-Tier Public Healthcare System in South Africa	Matuka et al. [14]	Cross-Sectional Study	South Africa	Southern Africa	Healthcare	Hospitals/clinics	Workplace	Facility
8	2023	Prevalence and Factors Associated with Tuberculosis Among the Mining Communities in Mererani, Tanzania	Mbuya et al. [15]	Cross-Sectional Study	Tanzania	East Africa	Mining	Mining	Workplace	Worksite
9	2024	The Influence of Humidity, Temperature, and Ventilation Area on the Incidence of Tuberculosis Among Farmers in Indonesia: A Case-Control Study	Adha et al. [16]	Analytical observational with a case control method	Indonesia	Southeast Asia	Agriculture	Residential	Workplace and house	Individual
10	2025	The Influence of Lighting, Floor Type, and Wall Type on Pulmonary Tuberculosis in Farmers	Adha et al. [17]	Analytical observational with a case control method	Indonesia	Southeast Asia	Agriculture	Residential	Workplace and house	Individual

DAIDS, Division of AIDS; NIAID, National Institute of Allergy and Infectious Diseases; NIH, National Institutes of Health; TB, tuberculosis.

Environmental exposures were assessed across different settings and spatial scales. Several studies focused specifically on workplace or facility-level exposures. However, some studies have also reported exposure to residential or mixed environments. The dwellings examined in the study of farmers are more accurately described as temporary living arrangements during work at the site. The environmental factors examined included residential ventilation, housing density, and housing conditions. These exposures are specifically mentioned to clarify the context of the living environment, which is still within their work area and poses risks related to tuberculosis.

The spatial scale of the included evidence ranged from individual-level assessments to worksite-, facility-, building-, and community-level evaluations. This variation highlights the geographical and environmental heterogeneity of the available evidence and should be considered when interpreting the relationship between environmental conditions and pulmonary TB among occupational populations.

### Ventilation and air circulation

Several studies consistently reported that poor ventilation and inadequate air circulation were strongly associated with increased TB risk among workers. Workplaces characterized by limited airflow, absence of mechanical ventilation systems, or poorly designed building structures were found to facilitate the accumulation of airborne infectious particles. Since pulmonary TB is primarily transmitted through airborne droplets containing *M. tuberculosis*, inadequate ventilation can increase the probability of transmission in enclosed workspaces.

### Workplace crowding and worker density

Workplace crowding was another commonly reported environmental risk factor across the included studies. High worker density within confined spaces increases the frequency and duration of close contact between individuals, which can facilitate airborne disease transmission. Several studies observed that workers operating in small or overcrowded indoor environments were more likely to develop TB than workers in less crowded settings. Overcrowding also often coincides with other environmental issues, such as poor ventilation and inadequate air circulation, further amplifying the risk of TB transmission.

### Dust and silica exposure

Occupational exposure to dust and silica particles was identified as one of the most significant environmental risk factors in industrial workplaces. Studies focusing on mining and construction sectors reported a higher incidence of TB among workers exposed to silica dust. Exposure to respirable silica particles can damage lung tissue and impair macrophage function, which weakens the body’s ability to control *M. tuberculosis* infection.

### Temperature and humidity

These factors may influence the survival of airborne microorganisms and the ventilation dynamics within indoor environments. Some studies suggested that poorly controlled temperature and humidity levels in indoor workplaces may be related to environmental conditions that allow airborne pathogens to persist longer.

### Indoor air quality

Indoor air quality emerged as an overarching factor that integrates several environmental determinants, including ventilation, dust exposure, humidity, and temperature. Workplaces with poor indoor air quality were more frequently associated with increased TB incidence among workers. Studies emphasized that inadequate environmental control measures, such as a lack of air filtration systems, insufficient ventilation, and high particulate concentrations, can create conditions conducive to airborne disease transmission.

## Discussion

### Principal findings

This scoping review synthesizes evidence from 10 studies examining the relationship between workplace environmental factors and the occurrence of TB among workers across different occupational sectors. The findings demonstrate that several physical environmental conditions in workplaces play an important role in influencing the risk of TB transmission. The most consistently reported determinants include inadequate ventilation, high worker density or overcrowding, exposure to dust and crystalline silica, and unfavorable indoor microclimatic conditions such as temperature and humidity. Among these factors, ventilation and air circulation emerged as the most frequently identified determinants of occupational TB risk. Workplaces characterized by limited air exchange and poor airflow were associated with higher concentrations of airborne infectious particles, thereby increasing the probability of disease transmission. In addition, occupational exposure to respirable dust, particularly crystalline silica, was repeatedly identified as a major environmental determinant of TB risk, especially among mining and industrial workers.

These findings reinforce the concept that TB transmission is not solely determined by individual-level risk factors, such as immune status or comorbidities, but is also strongly influenced by environmental and occupational conditions in which workers spend prolonged periods of time. Therefore, improving workplace environmental controls may represent an important intervention strategy for reducing occupational TB transmission.

### Comparison with global evidence

The findings of this review are consistent with growing global evidence indicating that environmental conditions in congregate settings strongly influence TB transmission dynamics. Several studies conducted in high-burden settings have demonstrated that ventilation rate and occupancy density are critical determinants of airborne TB transmission. For instance, a recent study examining congregate environments reported that lower ventilation rates combined with higher occupancy increased the estimated risk of infection transmission in indoor environments [18]. Similarly, research conducted in occupational settings such as hospitals, mines, and industrial workplaces has consistently reported increased TB risk among workers exposed to poorly ventilated indoor environments [19]. Healthcare workers face elevated occupational risk due to frequent exposure to infectious patients in clinical settings, particularly when infection prevention and control measures are inadequate.

In addition to ventilation-related risks, silica dust exposure has long been recognized as one of the strongest occupational risk factors for TB. A systematic review and meta-analysis demonstrated that workers with silicosis have a higher risk of developing pulmonary TB than unexposed populations, with pooled estimates indicating a several-fold increase in risk [7]. More recent studies continue to confirm this relationship. Evidence from occupational cohorts shows that workers exposed to silica dust, particularly in mining and construction sectors, have a substantially increased probability of developing TB infection or reactivation of latent TB [20].

These findings highlight that the interaction between occupational exposure and infectious disease transmission remains an important public health issue, especially in low- and middle-income countries where industrial safety regulations and environmental control measures may be insufficient.

### Biological mechanisms of TB transmission in the workplace

The spread of TB, an airborne infectious disease, in workplace settings is mainly caused by droplets carrying *M. tuberculosis*. When individuals with active pulmonary TB cough, sneeze, or speak, they release microscopic droplet nuclei containing the bacteria into the surrounding air. These droplet nuclei can remain suspended in the air for extended periods, particularly in poorly ventilated indoor spaces, and may be inhaled by individuals nearby [21]. Several workplace environmental conditions can facilitate this transmission process. Poor ventilation allows infectious aerosols to accumulate within indoor spaces, increasing the concentration of airborne bacteria and prolonging exposure among workers. Without adequate airflow, the dilution and removal of contaminated air become limited, creating conditions conducive to airborne disease spread.

Overcrowding or high worker density further amplifies this risk by increasing the frequency and duration of close interpersonal interactions. When multiple individuals share confined indoor environments for extended periods, the likelihood of inhaling infectious droplet nuclei increases substantially.

Occupational exposure to silica dust introduces an additional biological mechanism that increases susceptibility to TB infection [22]. Inhalation of crystalline silica particles damages pulmonary macrophages and impairs immune responses within the lungs. As a result, the body becomes less capable of controlling *M. tuberculosis* infection. This mechanism explains the well-known phenomenon of silicotuberculosis, in which individuals exposed to silica dust exhibit markedly higher TB incidence.

Furthermore, environmental factors such as temperature and humidity may influence the survival and persistence of airborne pathogens. Although the relationship between microclimate conditions and TB transmission remains complex, several studies suggest that poorly regulated indoor environments may support prolonged survival of airborne microorganisms, thereby increasing infection risk.

### Implications for occupational health policy

The findings of this scoping review have important implications for occupational health and workplace infection prevention policies. Given that many environmental risk factors identified in this study are modifiable, targeted workplace interventions could reduce the risk of TB transmission.

First, improving workplace ventilation systems should be prioritized, particularly in high-risk environments such as healthcare facilities, mines, construction sites, factories, and other enclosed workplaces. Increasing natural ventilation through windows and structural design, or installing mechanical ventilation systems, can help dilute and remove infectious aerosols from indoor spaces.

Second, administrative and environmental control measures should be implemented to reduce workplace crowding. This may include reorganizing workspaces, limiting the number of workers within confined areas, and adjusting shift schedules to minimize worker density.

Third, industries involving silica exposure, such as mining and construction, should adopt strict dust control strategies. These may include wet drilling techniques, improved ventilation in underground environments, and mandatory use of respiratory protective equipment for workers exposed to respirable dust particles.

In addition, occupational TB surveillance and screening programs should be strengthened, particularly in high-risk industries. Routine health monitoring and early detection strategies can help identify TB cases among workers and prevent further transmission within workplaces.

### Strengths and limitations

This scoping review has several strengths. First, the review applied a PRISMA-ScR methodology, ensuring a transparent and systematic process for identifying, screening, and selecting relevant studies. The inclusion of multiple international databases also helped to capture a wide range of relevant literature across different geographical regions.

Second, the review focused specifically on workplace environmental determinants, an area that remains relatively underexplored in TB research compared with individual-level risk factors. By synthesizing findings from different occupational sectors, this study provides a comprehensive overview of environmental conditions associated with occupational TB risk.

However, some limitations should also be acknowledged. Many of the included studies used cross-sectional designs, which limit the ability to establish causal relationships between workplace environmental exposures and TB outcomes. Longitudinal cohort studies would provide stronger evidence regarding temporal associations between exposure and disease development. Additionally, some studies relied on indirect proxies for environmental exposures, such as self-reported ventilation conditions or workplace characteristics, which may introduce measurement bias.

### Future research directions

Future research should aim to strengthen the evidence base regarding occupational environmental determinants of TB. First, prospective cohort studies are needed to evaluate long-term exposure to workplace environmental factors and their impact on TB incidence among workers. Second, more research is needed to quantify ventilation standards and air exchange thresholds that effectively reduce TB transmission in occupational settings. Advances in environmental monitoring technologies, such as real-time air quality sensors, could facilitate more accurate measurement of workplace conditions.

## Conclusion

This scoping review demonstrates that workplace physical environmental conditions play an important role in the transmission and occurrence of TB among workers. The reviewed studies consistently identified poor ventilation, inadequate air circulation, overcrowded workplaces, exposure to respirable dust and crystalline silica, and unfavorable indoor environmental conditions as major determinants of occupational TB risk. These factors may facilitate airborne transmission of *M. tuberculosis* and increase worker susceptibility, particularly in high-risk occupational sectors such as healthcare, mining, construction, and industry. The findings also suggest that silica exposure combined with inadequate ventilation may further amplify TB risk. Therefore, improving workplace environmental controls through better ventilation, dust suppression, workplace design, occupational health surveillance, and infection prevention programs is essential to reduce TB transmission in occupational settings.

## CRediT authorship contribution statement

**Mochammad Sholehhudin:** Writing – original draft, Formal analysis, Conceptualization. **Lutfi Fajar Nuraidah:** Formal analysis. **Aziz Fajar:** Data curation. **Tofan Agung Eka Prasetya:** Supervision. **Virasakdi Chongsuvivatwong:** Supervision. **Erwin Astha Triyono:** Data curation. **Sulvy Dwi Anggraini:** Data curation. **Faridha Cahyani:** Data curation. **Wihda Silvia Rahmah:** Project administration. **Allina Fiftiyani Nurjanah:** Project administration.

## Declaration of competing interest

The authors have no competing interests to declare. Mochammad Sholehhudin reports that administrative support was provided by Airlangga University. All authors declare that they have no known competing financial interests or personal relationships that could have appeared to influence the work reported in this paper.
